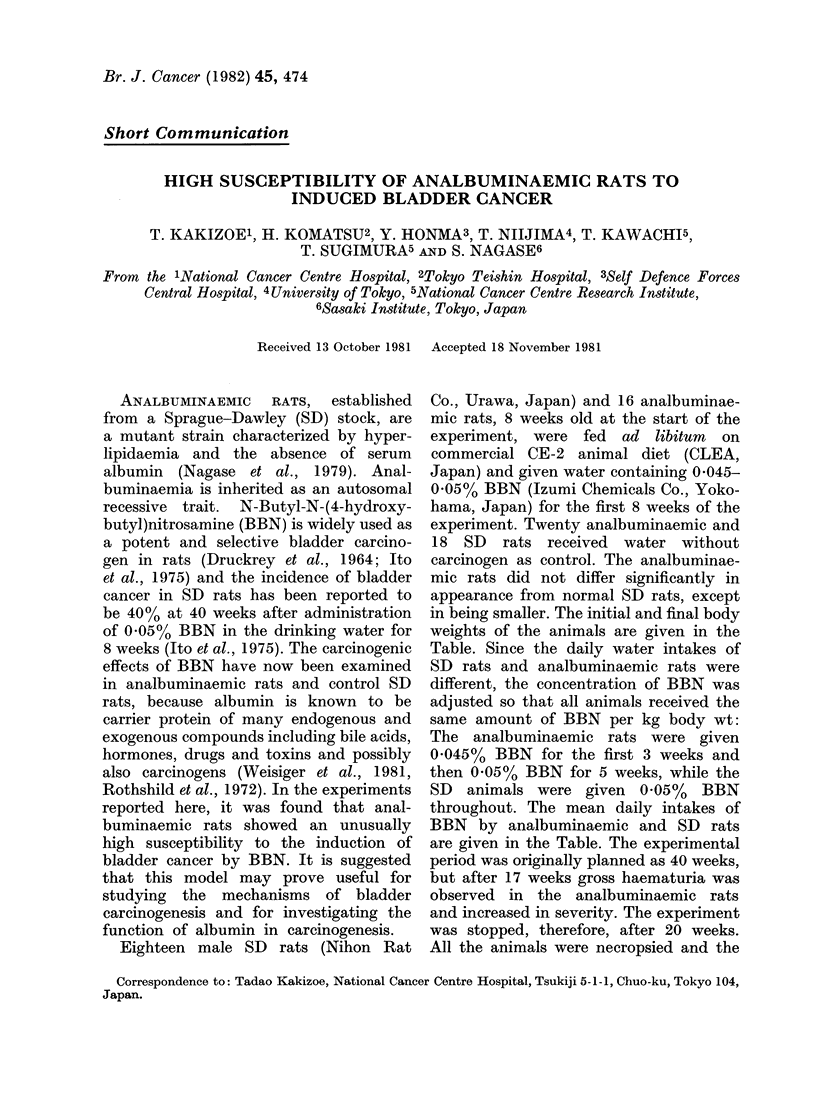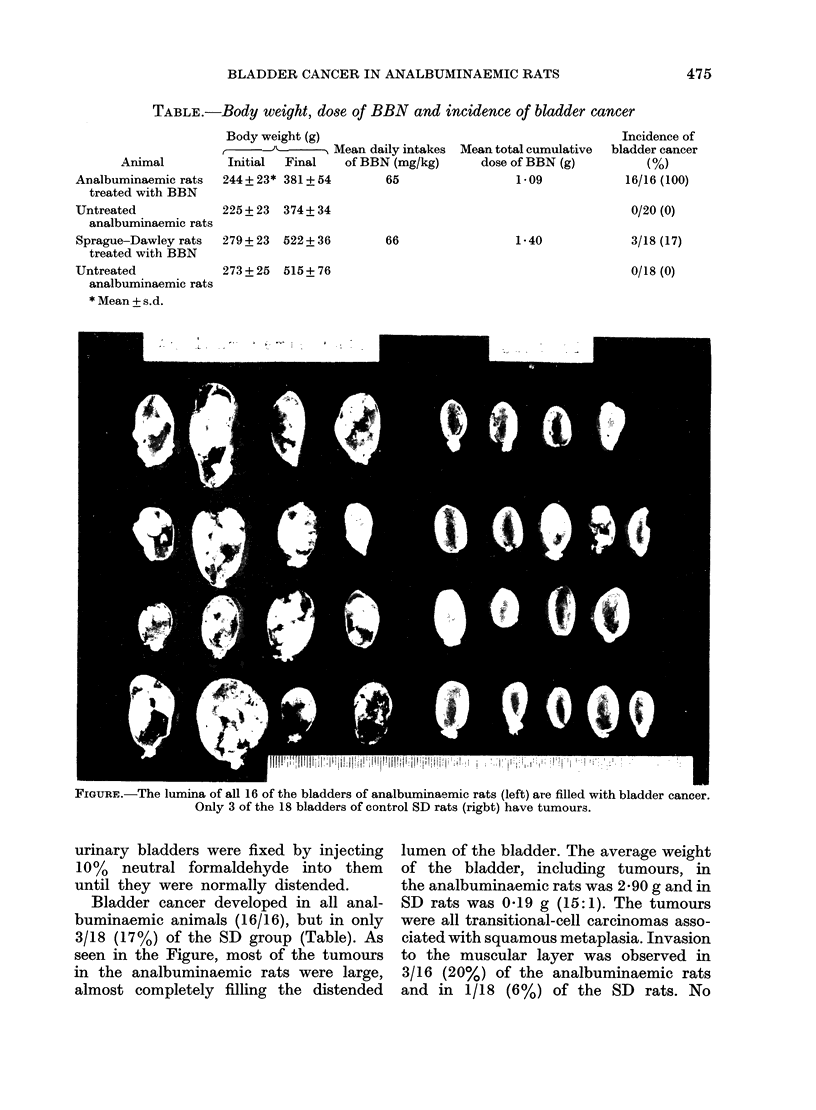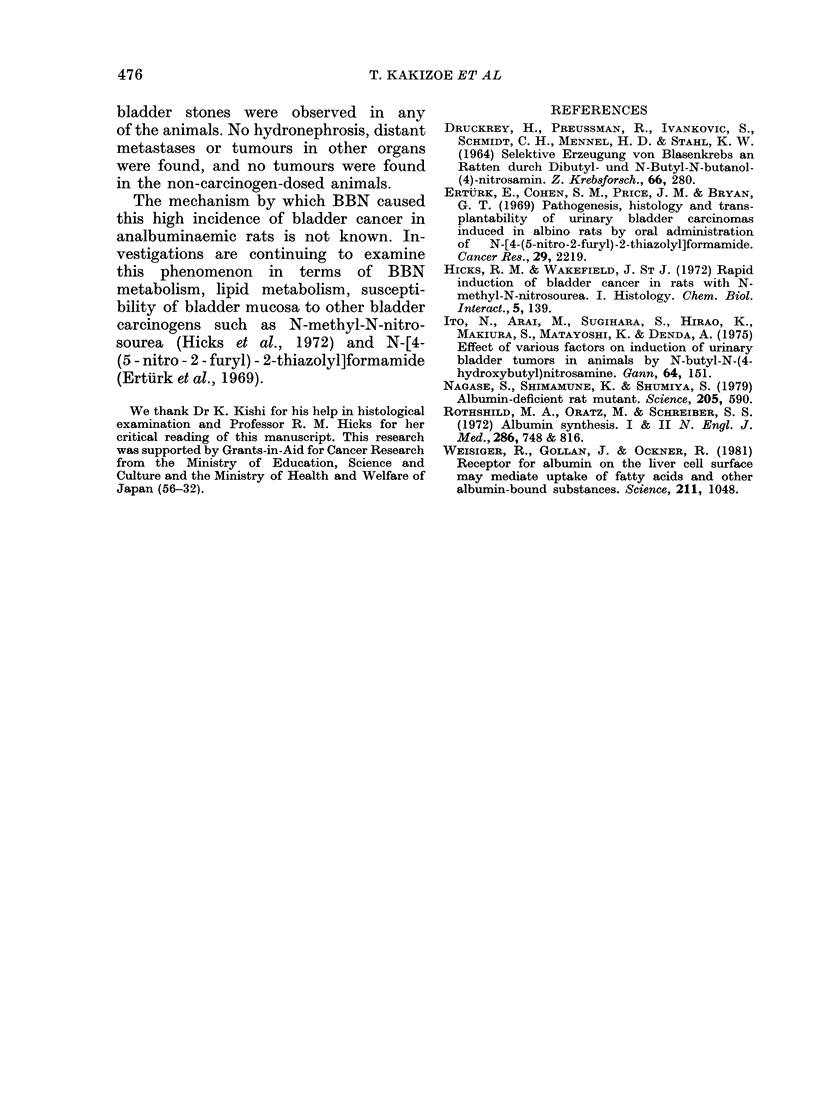# High susceptibility of analbuminaemic rats to induced bladder cancer.

**DOI:** 10.1038/bjc.1982.77

**Published:** 1982-03

**Authors:** T. Kakizoe, H. Komatsu, Y. Honma, T. Niijima, T. Kawachi, T. Sugimura, S. Nagase

## Abstract

**Images:**


Br. J. Cancer (1982) 45, 474

*Short Communication*

### HIGH SUSCEPTIBILITY OF ANALBUMINAEMIC RATS TO INDUCED BLADDER CANCER

T. KAKIZOE[1], H. KOMATSU[2], Y. HONMA[3], T. NIIJIMA[4], T. KAWACHI[5], T. SUGIMURA[5] AND S. NAGASE[6]

*From the [1]National Cancer Centre Hospital, [2]Tokyo Teishin Hospital, [3]Self Defence Forces Central Hospital, [4]University of Tokyo, [5]National Cancer Centre Research Institute, [6]Sasaki Institute, Tokyo, Japan*

Received 13 October 1981 Accepted 18 November 1981

ANALBUMINAEMIC RATS, established from a Sprague–Dawley (SD) stock, are a mutant strain characterized by hyperlipidaemia and the absence of serum albumin (Nagase *et al.*, 1979). Analbuminaemia is inherited as an autosomal recessive trait. N-Butyl-N-(4-hydroxybutyl)nitrosamine (BBN) is widely used as a potent and selective bladder carcinogen in rats (Druckrey *et al.*, 1964; Ito *et al.*, 1975) and the incidence of bladder cancer in SD rats has been reported to be 40% at 40 weeks after administration of 0·05% BBN in the drinking water for 8 weeks (Ito *et al.*, 1975). The carcinogenic effects of BBN have now been examined in analbuminaemic rats and control SD rats, because albumin is known to be carrier protein of many endogenous and exogenous compounds including bile acids, hormones, drugs and toxins and possibly also carcinogens (Weisiger *et al.*, 1981, Rothshild *et al.*, 1972). In the experiments reported here, it was found that analbuminaemic rats showed an unusually high susceptibility to the induction of bladder cancer by BBN. It is suggested that this model may prove useful for studying the mechanisms of bladder carcinogenesis and for investigating the function of albumin in carcinogenesis.

Eighteen male SD rats (Nihon Rat Co., Urawa, Japan) and 16 analbuminaemic rats, 8 weeks old at the start of the experiment, were fed *ad libitum* on commercial CE-2 animal diet (CLEA, Japan) and given water containing 0·045–0·05% BBN (Izumi Chemicals Co., Yokohama, Japan) for the first 8 weeks of the experiment. Twenty analbuminaemic and 18 SD rats received water without carcinogen as control. The analbuminaemic rats did not differ significantly in appearance from normal SD rats, except in being smaller. The initial and final body weights of the animals are given in the Table. Since the daily water intakes of SD rats and analbuminaemic rats were different, the concentration of BBN was adjusted so that all animals received the same amount of BBN per kg body wt: The analbuminaemic rats were given 0·045% BBN for the first 3 weeks and then 0·05% BBN for 5 weeks, while the SD animals were given 0·05% BBN throughout. The mean daily intakes of BBN by analbuminaemic and SD rats are given in the Table. The experimental period was originally planned as 40 weeks, but after 17 weeks gross haematuria was observed in the analbuminaemic rats and increased in severity. The experiment was stopped, therefore, after 20 weeks. All the animals were necropsied and the

Correspondence to: Tadao Kakizoe, National Cancer Centre Hospital, Tsukiji 5-1-1, Chuo-ku, Tokyo 104, Japan.

TABLE.—*Body weight, dose of BBN and incidence of bladder cancer*

| Animal | Body weight (g) Initial | Body weight (g) Final | Mean daily intakes of BBN (mg/kg) | Mean total cumulative dose of BBN (g) | Incidence of bladder cancer (%) |
|---|---|---|---|---|---|
| Analbuminaemic rats treated with BBN | 244 ± 23* | 381 ± 54 | 65 | 1·09 | 16/16 (100) |
| Untreated analbuminaemic rats | 225 ± 23 | 374 ± 34 | | | 0/20 (0) |
| Sprague–Dawley rats treated with BBN | 279 ± 23 | 522 ± 36 | 66 | 1·40 | 3/18 (17) |
| Untreated analbuminaemic rats | 273 ± 25 | 515 ± 76 | | | 0/18 (0) |

* Mean ± s.d.

FIGURE.—The lumina of all 16 of the bladders of analbuminaemic rats (left) are filled with bladder cancer. Only 3 of the 18 bladders of control SD rats (rigbt) have tumours.

urinary bladders were fixed by injecting 10% neutral formaldehyde into them until they were normally distended.

Bladder cancer developed in all analbuminaemic animals (16/16), but in only 3/18 (17%) of the SD group (Table). As seen in the Figure, most of the tumours in the analbuminaemic rats were large, almost completely filling the distended lumen of the bladder. The average weight of the bladder, including tumours, in the analbuminaemic rats was 2·90 g and in SD rats was 0·19 g (15:1). The tumours were all transitional-cell carcinomas associated with squamous metaplasia. Invasion to the muscular layer was observed in 3/16 (20%) of the analbuminaemic rats and in 1/18 (6%) of the SD rats. No bladder stones were observed in any of the animals. No hydronephrosis, distant metastases or tumours in other organs were found, and no tumours were found in the non-carcinogen-dosed animals.

The mechanism by which BBN caused this high incidence of bladder cancer in analbuminaemic rats is not known. Investigations are continuing to examine this phenomenon in terms of BBN metabolism, lipid metabolism, susceptibility of bladder mucosa to other bladder carcinogens such as N-methyl-N-nitrosourea (Hicks *et al.*, 1972) and N-[4-(5-nitro-2-furyl)-2-thiazolyl]formamide (Ertürk *et al.*, 1969).

We thank Dr K. Kishi for his help in histological examination and Professor R. M. Hicks for her critical reading of this manuscript. This research was supported by Grants-in-Aid for Cancer Research from the Ministry of Education, Science and Culture and the Ministry of Health and Welfare of Japan (56–32).

REFERENCES

Druckrey, H., Preussman, R., Ivankovic, S., Schmidt, C. H., Mennel, H. D. & Stahl, K. W. (1964) Selektive Erzeugung von Blasenkrebs an Ratten durch Dibutyl- und N-Butyl-N-butanol-(4)-nitrosamin. *Z. Krebsforsch.*, **66**, 280.

Ertürk, E., Cohen, S. M., Price, J. M. & Bryan, G. T. (1969) Pathogenesis, histology and transplantability of urinary bladder carcinomas induced in albino rats by oral administration of N-[4-(5-nitro-2-furyl)-2-thiazolyl]formamide. *Cancer Res.*, **29**, 2219.

Hicks, R. M. & Wakefield, J. St J. (1972) Rapid induction of bladder cancer in rats with N-methyl-N-nitrosourea. I. Histology. *Chem. Biol. Interact.*, **5**, 139.

Ito, N., Arai, M., Sugihara, S., Hirao, K., Makiura, S., Matayoshi, K. & Denda, A. (1975) Effect of various factors on induction of urinary bladder tumors in animals by N-butyl-N-(4-hydroxybutyl)nitrosamine. *Gann*, **64**, 151.

Nagase, S., Shimamune, K. & Shumiya, S. (1979) Albumin-deficient rat mutant. *Science*, **205**, 590.

Rothshild, M. A., Oratz, M. & Schreiber, S. S. (1972) Albumin synthesis. I & II *N. Engl. J. Med.*, **286**, 748 & 816.

Weisiger, R., Gollan, J. & Ockner, R. (1981) Receptor for albumin on the liver cell surface may mediate uptake of fatty acids and other albumin-bound substances. *Science*, **211**, 1048.